# Development of a novel target module redirecting UniCAR T cells to Sialyl Tn-expressing tumor cells

**DOI:** 10.1038/s41408-018-0113-4

**Published:** 2018-08-22

**Authors:** L. R. Loureiro, A. Feldmann, R. Bergmann, S. Koristka, N. Berndt, C. Arndt, J. Pietzsch, C. Novo, P. Videira, M. Bachmann

**Affiliations:** 1 UCIBIO, Departamento Ciências da Vida, Faculdade de Ciências e Tecnologia, Universidade Nova de Lisboa, Lisboa, Portugal; 2 Helmholtz-Zentrum Dresden-Rossendorf (HZDR), Institute of Radiopharmaceutical Cancer Research, Dresden, Germany; 3 UniversityCancerCenter (UCC) Dresden, Tumor Immunology, ‘Carl Gustav Carus’ Technische Universität Dresden, Dresden, Germany; 4 German Cancer Consortium (DKTK), German Cancer Research Center (DKFZ), partner site Dresden, Heidelberg, Germany; 5 Faculty of Chemistry and Food Chemistry, School of Science, Technische Universität Dresden, Dresden, Germany; 6 UEIPM, Instituto de Higiene e Medicina Tropical, Universidade NOVA de Lisboa, Lisboa, Portugal; 7 CDG & Allies, Professional and Patient Association International Network (PPAIN), Caparica, Portugal; 8 National Center of Tumor Diseases (NCT), partner site Dresden, Dresden, Germany

Genetic engineering of T cells with chimeric antigen receptors (CARs) that specifically target tumor cells is emerging as a promising new treatment for a wide range of cancers^1^. CARs that are currently used in the clinic consist of three domains: (i) a tumor-specific extracellular domain that usually comprises the variable heavy and light chain sequences of a monoclonal antibody (mAb) in the form of a recombinant single chain fragment variable (scFv), providing specificity to target an antigen on the surface of tumor cells, (ii) a transmembrane domain that is commonly derived from the CD8 or CD28 receptor, and (iii) intracellular signaling domains consisting of activation motifs of immune receptors for signal transduction. Based on the number of different intracellular signaling domains first, second, and third generation CARs can be categorized^2^. The tremendous potential of CAR technology has been proven by the clinical success of CD19-specific CAR T cells^3^. However, safety issues are the most limiting features for a broader application of the current CAR T cell technology in particular for the treatment of solid tumors. Mainly on-target/off-tumor toxicities against healthy tissues and excessive on-target/on-tumor reactions leading to life-threatening cytokine release syndrome (CRS) or tumor lysis syndrome represent currently the most critical side effects^4^. For instance CD19 expression is not restricted to leukemic cells but also present on healthy B cells. Therfore, treatment with anti-CD19 CARs causes long-term B cell aplasia in patients. Absence of B cells can be easily treated by intravenous immunoglobulin administration; however, targeting other tumor-associated antigens (TAAs) such on-target/off-tumor effects may not be acceptable^5^.

To overcome such limitations the previously described universal modular antibody-based platform technology named UniCAR may be used^6^. In this system, the cross-linkage of effector cells and target cells is not directly mediated by the extracellular domain of the CAR but via a complex of two components (Fig. 1a). The first component is a UniCAR-engineered T cell with a binding domain against a peptide epitope (E5B9, UniCAR tag) derived from the nuclear protein La/SS-B which is not accessible on the surface of intact living cells. The second component is a target module (TM) composed of a binding moiety against a TAA of choice. The epitope recognized by the UniCAR is fused to this TM in order to redirect the otherwise inert UniCAR-expressing T cells (UniCAR T cells) to the respective target cells^6^. Moreover, UniCAR-expressing T cells (UniCAR T cells) can reversibly be armed with one or multiple TMs. Upon eradication of all tumor cells or severe side effects occur, inactivation of the UniCAR T cells can be achieved simply by ceasing infusion of the TM. Thus, both activity and potential adverse effects of UniCAR T cells may be tunable by dosing TM infusions. According to this idea a series of UniCAR TMs with suitable pharmacokinetic features have recently been described^6–8^.

**Fig. 1:**
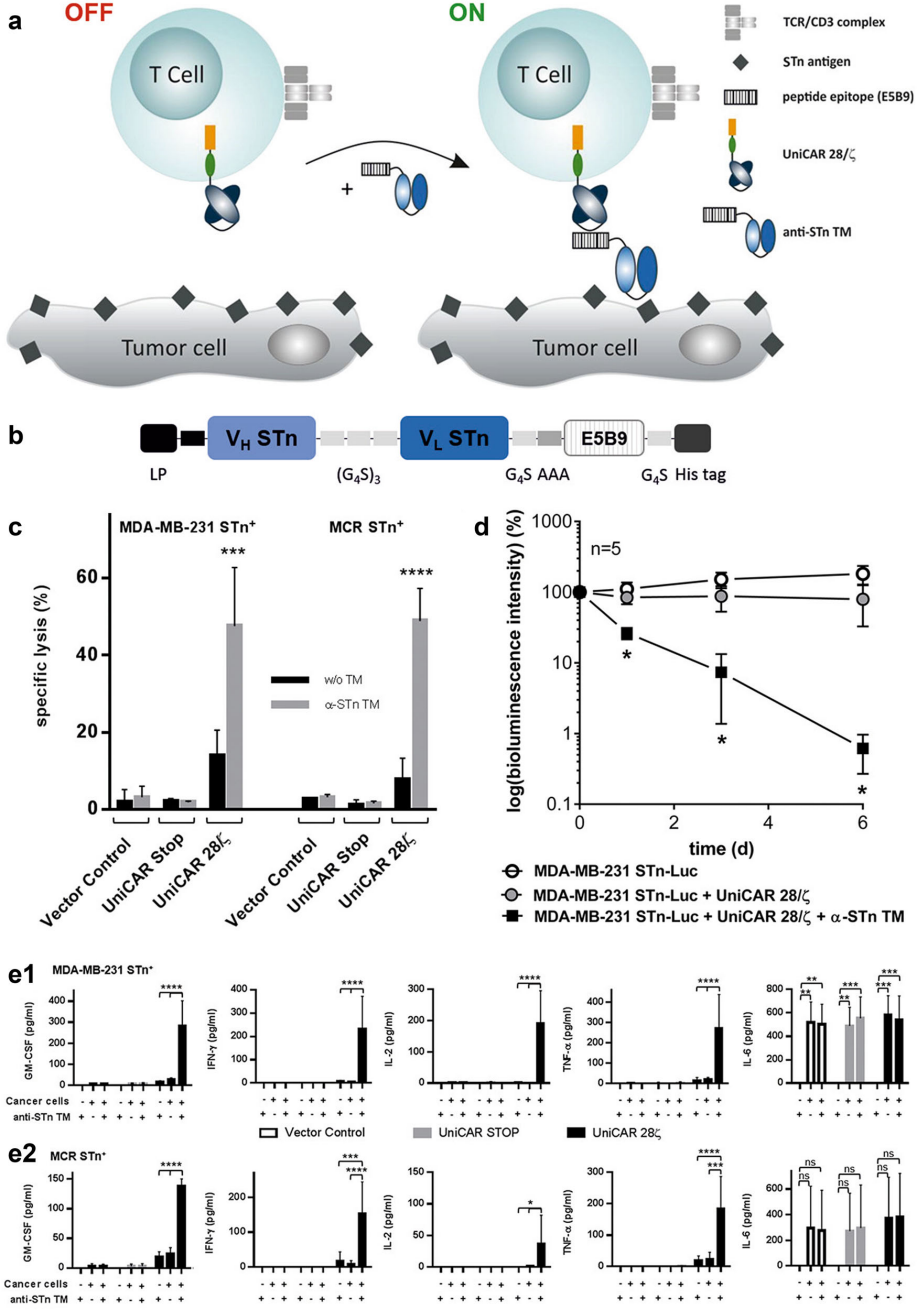
Development of the anti-STn TM, subsequent TM dependent killing in vitro and in vivo, and cytokine release assays. **a** The UniCAR system consists of T cells genetically engineered with UniCARs, containing a humanized scFv derived from the anti-La mAb E5B9 fused to the transmembrane domain of human CD28. Intracellularly, the construct comprises signaling domains of CD28 and CD3ζ. In the absence of an antigen-specific TM, UniCAR T cells are unable to bind to target cells^6^. However, in the presence of an anti-STn TM a complex is formed via the UniCAR epitope (E5B9), endorsing binding of UniCAR T cells to STn-expressing cancer cells. **b** The anti-STn TM comprises the variable domains derived from the mAb L2A5 arranged in V_H_–V_L_ orientation separated by a (G_4_S)_3_ peptide linker. The UniCAR epitope was fused C-terminally and is flanked by two spacer peptides (N-terminal spacer: AAA; C-terminal spacer: G_4_S). To allow protein purification and detection, the TM was tagged with 6xhis residues at the C-terminus. In addition, an N-terminal leader peptide (LP) was added to ensure secretion of the recombinant protein into the cell culture supernatant^15^. **c** T cell-mediated tumor cell killing was measured using standard chromium release assays. MDA-MB-231 and MCR STn-expressing cell lines were incubated with T cells engrafted with either the vector control (vector backbone encoding only the EGFP marker protein), UniCAR STOP construct (lacking intracellular signaling domains) or UniCAR signaling construct (UniCAR 28/ζ). Both tumor cell lines were cultivated with the respective genetically engineered T cells at an effector to target (E:T) ratio of 5:1 in the presence or absence of 80 nM anti-STn TM for 24 h ^7^. Mean specific lysis and SD of three independent T cell donors are shown. **d** MDA-MB-231 STn-expressing cells were transduced to express firefly luciferase resulting in MDA-MB-231 STn-Luc cells. Per mouse, either 1.5 × 10^6^ tumor cells alone, mixed with 1 × 10^6^ UniCAR 28/ζ T cells or mixed with 1 × 10^6^ UniCAR 28/ζ T cells and 10 µg of anti-STn TM were injected subcutaneously into female NMRI-Foxn1^nu^/Foxn1^nu^ mice resulting in three groups of animals each consisting of five mice. Quantitative evaluation of the luminescence imaging of anesthetized mice at day 0 and followed at day 1, 3, and 6. **e1,e2** Genetically engineered UniCAR T cells were incubated for 24 h in the presence or absence of MDA-MB-231 STn^+^ (**e1**) or MCR STn^+^ (**e2**) cells as well as in the presence or absence of 80 nM anti-STn TM. As controls, the same conditions were performed using T cells transduced with the vector control or with the UniCAR STOP. Cell culture supernatants from three individual donors were collected and analyzed using MACSPlex Cytokine 12 Kit and MACSQuantify® software according to the manufacturer’s instructions. SD of mean cytokine values of three independent T cell donors are shown. Statistical analysis was performed using one-way or two-way ANOVA with Bonferroni multiple-comparison test (ns = not significant; *p < 0.1; **p < 0.01; ***p < 0.001, and ****p < 0.0001)

Sialyl-Tn (STn, Neu5Acα2,6GalNAcα-O-Ser/Thr) is a truncated *O*-glycan resulting from the early sialylation with α2,6-linked sialic acid (*N*-acetyl-neuraminic acid or Neu5Ac) of the Tn antigen, a *N*-acetyl-galactosamine *O*-linked to a serine or threonine residue in a polypeptide chain. STn modulates a malignant phenotype associated with a poor prognosis in cancer patients concerning tumor progression, immune invasion, and metastasis. STn is overexpressed in more than 80% of human carcinomas, such as bladder, ovarian, colon, breast, and prostate cancers but rarely found in healthy tissues^9^. Therefore, in the past decades attempts to develop effective cancer therapies targeting STn have been performed^10^. Yet, so far there is no clinical approved therapy. Novel technologies including CAR T cell therapies may have the potential to develop more efficient cancer therapies against STn. We therefore tried to establish a UniCAR system for retargeting of STn positive tumor cells. As some cross-reactivities of anti-STn mAbs with immune cells were described^11^, we first wanted to identify the anti-STn paratope most promising for such an approach. For this reason, we compared the binding of the three anti-STn mAbs L2A5^12^, B72.3, and 3F1 (also known as HB-STn-1)^13^ to subpopulations of immune cells using flow cytometry (Supplementary Fig. 1). While none of these anti-STn mAbs stained NK cells (data not shown), the anti-STn mAbs B72.3 and 3F1 bound to CD4^+^ and CD8^+^ T cells but not to B cells. In contrast, the anti-STn mAb L2A5 showed some binding activity to B cells but only a weak binding to a subpopulation of CD4^low^ cells (Supplementary Fig. 1). According to these data, an immunotherapeutic approach based on a CAR T cell derived from any of these anti-STn mAbs might harbor the risk of fratricide of T- and/or B lymphocytes. Bearing in mind that anti-CD19 CAR T cells causing B cell elimination is clinically manageable, an anti-STn CAR leading to the loss of B cells should be less risky. Thus, the mAb L2A5 appeared as the most suitable for our purpose. Moreover, a switchable CAR T cell approach such as the UniCAR technology should be preferable over conventional CAR T cells to avoid long-lasting or permanent, perhaps life-threatening loss of distinct immune cell subpopulations. In light of all these data, we decided to develop a TM based on the anti-STn mAb L2A5 for retargeting of UniCAR T cells to STn-expressing tumor cells via the switchable UniCAR platform.

As schematically represented in Fig. 1b, the anti-STn TM was generated by fusing the UniCAR epitope to the anti-STn scFv (Supplementary Materials and Methods). Following established protocols, a permanent cell line expressing and secreting the TM was established (Supplementary Materials and Methods). Purification of the TM from cell culture supernatants was accomplished by Ni-NTA affinity chromatography. The purified TM was further analyzed by SDS-PAGE and immunoblotting confirming the correct molecular weight and purity essential for use in further assays^7,14^. Next, we confirmed the binding capability of the anti-STn TM and estimated its binding affinity. For this purpose, the binding of the TM to the breast cancer cell line MDA-MB-231 and the bladder carcinoma cell line MCR was assessed by flow cytometry. As STn expression is downregulated in cultured tumor cells these cells were genetically manipulated to overexpress the STn antigen^12^. Staining with B72.3 and L2A5 mAbs served as positive controls (Supplementary Fig. 2A, left and middle panels). According to these results the anti-STn TM binds to STn-expressing cells. For the binding of the anti-STn TM to STn^+^ MDA-MB-231 a *K*_D_ value of 73.61 nM was estimated (Supplementary Fig. 2B). As first functional evidence, we searched for a TM-dependent and STn-specific activation of UniCAR T cells. Indeed, UniCAR T cells co-cultured with either the STn^+^ MDA-MB-231 (Supplementary Fig. 3A) or STn^+^ MCR (Supplementary Fig. 3B) cell line were activated in a TM-dependent and target-specific manner. In order to analyze tumor cell lysis mediated by STn-redirected UniCAR T cells, chromium release assays were performed using MDA-MB-231 and MCR STn-expressing cell lines (Fig. 1c). As shown in Fig. 1c, incubation of UniCAR 28/ζ T cells with the anti-STn TM leads to an efficient lysis of both STn^+^ cancer cell lines. In contrast, in the absence of the TM tumor cells were not attacked by co-cultured UniCAR 28/ζ T cells. Besides, no killing was observed for vector control- and UniCAR STOP-transduced T cells neither in the presence nor absence of the TM. Moreover, MDA-MB-231 and MCR cancer cell lines lacking the expression of the STn antigen were not attacked (data not shown). EC_50_ values of 12.2 and 25.0 nM were estimated for STn^+^ MDA-MB-231 and STn^+^ MCR cancer cells, respectively. Taken together, these data demonstrate that killing of STn-expressing cells by UniCAR T cells is target specific and occurs in a strictly TM-dependent manner. For analysis of cytokines released during retargeting of tumor cells with the UniCAR T cells in the presence or absence of the ant-STn TM we used a multiplex assay (Supplementary Materials and Methods). This assay allows the simultaneous detection and quantification of the cytokines GM-CSF, IFN-α, IFN-γ, IL-2, IL-4, IL-5, IL-6, IL-9, IL-10, IL-12, IL-17A, and TNF-α. Except for GM-CSF, IFN-γ, IL-2, IL-6, and TNF-α, no cytokines were detected at significant concentrations (Fig. 1e). Cytokines were only detected for UniCAR T cells co-cultured with STn-expressing cancer cells in the presence of the anti-STn TM, except for the cytokine IL-6. In this particular case, IL-6 was present in all conditions including cancer cells (Fig. 1e, rightmost plots). These results suggest that IL-6 is most probably secreted by the STn-expressing cancer cell lines themselves. Furthermore, cytokines were not detected in supernatants from negative controls and from UniCAR T cells in the absence of TM and/or cancer cells. The killing capability of UniCAR T cells armed with the anti-STn TM can also be confirmed using a xenograft tumor mouse model. For that, STn^+^ MDA-MB-231 cells were transduced with firefly luciferase (MDA-MB-231 STn-Luc cells) and mixed with UniCAR 28/ζ T cells in the presence of anti-STn TM. Tumor cells alone or UniCAR 28/ζ T cells in the absence of anti-STn TM served as negative controls. The respective mixtures were injected subcutaneously into female NMRI-Foxn1^nu^/Foxn1^nu^ mice and tumor growth was monitored by bioluminescence imaging for 6 days (Fig. 1d). In the presence of the anti-STn TM UniCAR 28/ζ T cells completely inhibited tumor growth. Conversely, in the absence of the anti-STn TM UniCAR 28/ζ T cells could not hinder tumor growth. In order to show that the anti-STn TM fulfills the pharmacological requirements of a TM useful for the UniCAR system, we estimated its pharmacokinetic behavior. For this purpose, the anti-STn TM was conjugated with the chelator NODAGA (NODAGA-anti-STn TM) and further labeled with the PET isotope ^64^Cu^2+^ ([^64^Cu]Cu-NODAGA-anti-STn TM). Figure 2 summarizes the biodistribution of the [^64^Cu]Cu-NODAGA-anti-STn TM as either activity concentration (Fig. 2a) or percentage of the total activity of the injected dose (Fig. 2b) for a total of four MDA-MB-231 STn-Luc tumor-bearing mice at 2 h after single intravenous injection. The compound was equally eliminated via kidneys (12.6 ± 4.0 %ID) and liver (11.8 ± 2.1 %ID). However, the activity concentration in the kidneys (9.19 ± 2.84 SUV) was higher than the liver value (2.08 ± 0.36 SUV). The tumor to muscle ratio, representing the target to background ratio, was higher compared to tumor to blood ratio presenting values of 5.3 ± 1.3 and 1.6 ± 0.3, respectively (Fig. 2c). In addition, small animal PET was performed using the radiolabeled anti-STn TM. For that, MDA-MB-231 STn-Luc tumors were established on the right hind flank of experimental mice. The PET images (Fig. 2d, e) show that [^64^Cu]Cu-NODAGA-anti-STn TM clearly accumulates at the tumor. In order to assess the pharmacokinetic behavior of the novel anti-STn TM in more detail, time-activity curves (TAC) of regions of interest (ROI) were derived from the PET studies of MDA-MB-231 STn-Luc tumor-bearing mice. Representative data shown in Fig. 2f were derived from a dynamic PET study collected over 2 h and a static measurement 13 h after a single intravenous injection of ~7 MBq [^64^Cu]Cu-NODAGA-anti-STn TM. Based on these data, target to background curves were calculated as tumor to muscle and tumor to blood ratios (Fig. 2g). The curves show that the fastest activity clearance was observed in the kidneys with a half-life of 3.1 min (95% confidence interval [c.i.] 1.8-9.6 min), followed by the liver with half-life of 7.8 min (c.i. 7.0–8.8 min), muscle 11.7 min (c.i. 9.2–16.1 min) and the tumor with the longest half-life of 110 min (c.i. 86–154 min). The curve shapes of the tumor to muscle and tumor to blood ratios are continuously increasing. Starting with a half hour after injection the curves were nearly linearly. In summary, these data show that the anti-STn is rapidly eliminated and thus might allow a rapid on and off switch of the UniCAR system in case severe side effects occur.

**Fig. 2:**
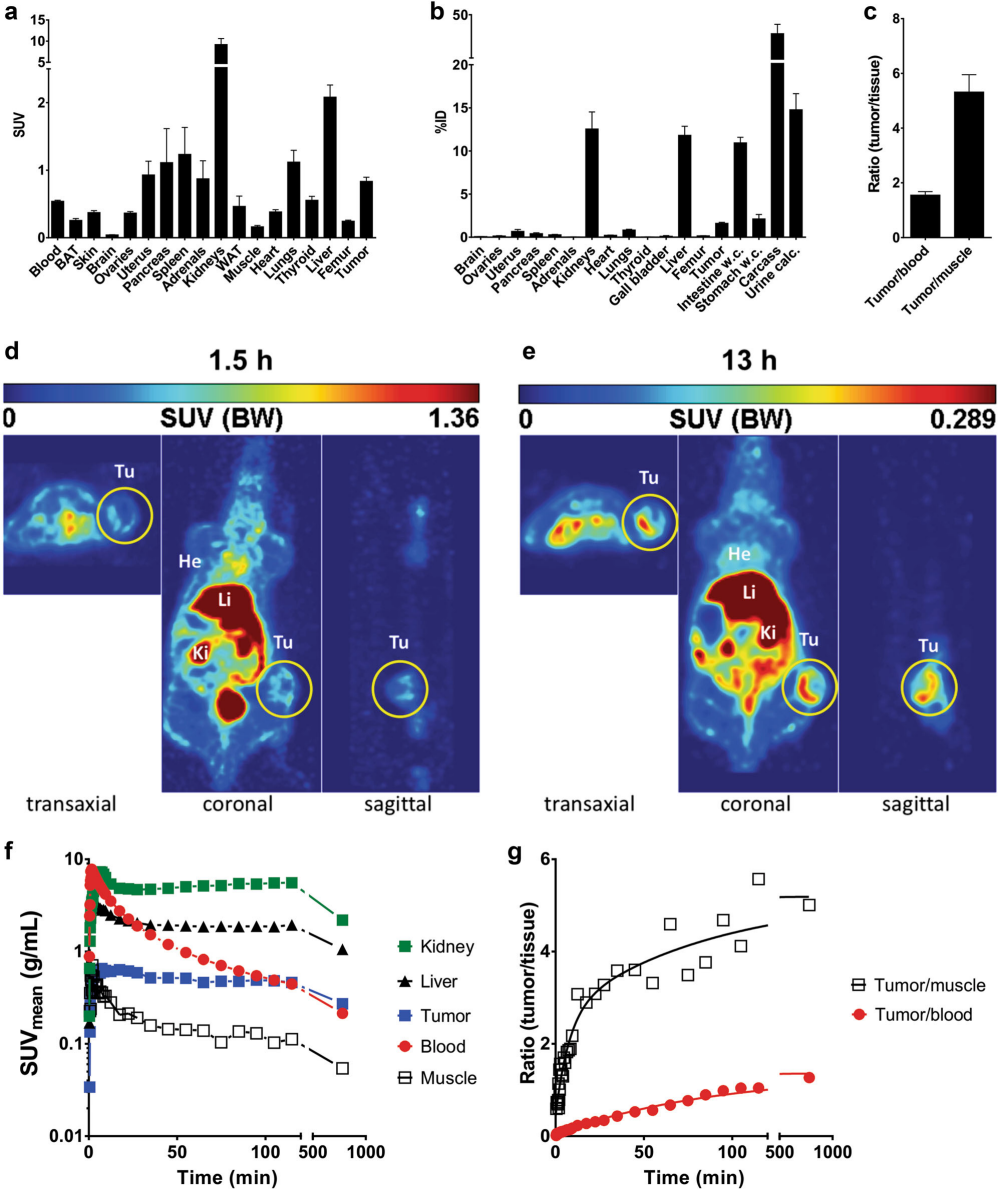
Biodistribution, imaging, and time-activity curves of [^64^Cu]Cu-NODAGA-anti-STn TM in STn positive tumor bearing mice. [^64^Cu]Cu-NODAGA-anti-STn TM was developed according to standard procedures^7^ and intravenously injected in NMRI-Foxn1^nu/nu^ MDA-MB-231 STn-Luc tumor-bearing mice with 3–4 weeks of tumor growth (1.5 × 10^6^ cells, *n* = 4). Two hours after injection, the biodistribution of the [^64^Cu]Cu-NODAGA-anti-STn TM was measured and shown as activity concentration (SUV) (**a**) or percentage of the total activity of the injected dose %ID (**b**) in the selected organs and tissues. Target to background ratios including tumor to muscle-, and tumor to blood ratios are shown in (**c**). Values are represented as mean ± SEM. **d**, **e** Representative orthogonal images of a PET study at 1.5 h (**d**) and 13 h (**e**) after single intravenous injection of ~7 MBq [^64^Cu]Cu-NODAGA-anti-STn TM. **f** Time activity concentration curves of representative organs of an NMRI-Foxn1^nu/nu^ mouse bearing an MDA-MB-231 STn-expressing tumor derived from a dynamic PET study over 2 h and a static at 13 h after single intravenous injection of ~7 MBq [^64^Cu]Cu-NODAGA-anti-STn TM. **g** Resulting target to background curves calculated as tumor to muscle and tumor to blood ratios

Taken together, the data presented here reveal that UniCAR T cells can be efficiently and safely re-directed to STn-positive cancer cells using the anti-STn TM based on the mAb L2A5. Retargeting of UniCAR T cells results in effective eradication of STn-expressing breast- and bladder-associated tumor cells both in vitro and in vivo. Therefore, the UniCAR platform represents a promising approach for targeting malignant STn-expressing cells in different types of cancer in a controlled manner. Moreover, the anti-STn TM also demonstrates high potential as diagnostic tool for PET-imaging of STn-positive tumors.

## Electronic supplementary material

Supplementary Materials and Methods

Supplementary Figure 1

Figure Legend Suppl. Fig. 1

Supplementary Figure 1 Text summary

Supplementary Figure 2

Figure Legend Suppl. Fig. 2

Supplementary Figure 2 Text summary

Supplementary Figure 3

Figure Legend Suppl. Fig. 3

Supplementary Figure 3 Text summary
