# Supplementary material for: Development of a novel target module redirecting UniCAR T cells to Sialyl Tn-expressing tumor cells
Source: Blood Cancer J. 2018 Aug 22;8(9):81. doi: 10.1038/s41408-018-0113-4 (PMC6127150; doi:10.1038/s41408-018-0113-4)
Supplement: Supplementary file 1 — Supplementary Materials and Methods [file 41408_2018_113_MOESM1_ESM.docx]

**Development of a novel target module redirecting UniCAR T cells to Sialyl Tn-expressing tumor cells**

Liliana Loureiro^1,2,3^, Anja Feldmann^2^, Ralf Bergmann^2^, Stefanie Koristka^2^, Nicole Berndt^2,4^, Jens Pietzsch^2,5^, Carlos Novo^1,6^, Paula Videira^1,6^, Michael Bachmann^2,3,4,8^

### MATERIALS AND METHODS

#### Cell lines

Human bladder cancer cell line transfected variants MCR NC and MCR STn were generated as previously described^1^. The human breast cancer line MDA-MB-231 WT and transfectants for human ST6GalNAc-I were also previously generated as described by Julien *et al*^2^. For all experiments, cells were harvested from culture flasks using Trypsin-EDTA in sterile phosphate-buffered saline (PBS). Human Embryonic Kidney cells HEK293T (ATCC CRL-11268) were also used and all the above-mentioned cell lines were cultured in Dulbecco’s modified Eagle medium (DMEM), supplemented with 10% (v/v) FBS, 2 mM L-glutamine, 100 U/ml of penicillin and 100 μg/ml of streptomycin (Biochrom GmbH, Berlin, Germany). The 3T3 (ATCC CRL-1658) fibroblast cells were used for anti-STn TM production and cultured in complete RPMI 1640 medium with 10% FCS, 100 U/ml penicillin and 100 µg/ml streptomycin, 2 mM N-acetyl-L-alanyl-L-glutamine, 1% non-essential amino acids and 1 mM sodium pyruvate (Biochrom GmbH, Berlin, Germany). To perform the *in vivo* analysis MDA-MB-231 STn cell line was further transduced with an open reading frame encoding the firefly luciferase gene as described previously and named MDA STn Luc^+^ (ref. 3). All cell lines were maintained at 37^o^C in a humidified atmosphere of 5% CO_2_.

#### Construction and expression of recombinant antibodies

The novel anti-STn TM was derived from mAb L2A5 (ref. 27) from which the variable regions were sequenced and further cloned into the lentiviral vector p6NST50 as previously described^4^. Briefly, the sequences encoding its variable light and heavy chain were connected by three times repeat of G_4_S and fused to the UniCAR tag followed by a 6xhis tag. Stable recombinant TM producing 3T3 cell lines were established by lentiviral gene transfer and recombinant proteins were purified from cell supernatants via Ni-NTA affinity chromatography followed by determination of protein concentration and purity through SDS–polyacrylamide gel electrophoresis and immunoblotting as previously described^4,5^. Binding properties of the novel TMs were analyzed by flow cytometry analysis as described previously^6^.

#### Generation of UniCAR vectors

Cloning of the humanized anti-La mAb E5B9 derived UniCAR scFv, generation of the hinge, transmembrane and signaling domain of the UniCAR is described in detail^7^. A peptide epitope of 18 aa (7B6-tag)^8,9^ followed by an additional G_4_S linker were inserted between the UniCAR scFv and the CD28 coding region. The mAb 7B6 was generated by standard hybridoma fusion technique and identified to specifically bind to the introduced epitope sequence^9^. Both epitopes are parts of the nuclear autoantigen La/SS-B. The CAR signaling and STOP constructs were subsequently cloned into the lentiviral vector p6NST60 (ref. 34). The open reading frames (*orf*) of the CAR constructs were fused to an EGFP *orf* separated by a 2pA protease site derived from the *Thosea asigna* virus that promotes the translation of CAR and EGFP as a fusion protein from a single mRNA in modified T cells. The fusion protein is proteolytically cleaved after translation^10^.

#### Isolation of peripheral blood mononuclear cells (PBMCs), T cell subpopulations and lentiviral transduction

Isolation of primary human T cells from peripheral blood mononuclear cells (PBMCs) out of buffy coats (supplied by German Red Cross, Dresden, Germany) or from fresh blood of healthy donors was performed as described previously^5^. The study including the consent form was approved by the local ethics committee of the university hospital of the medical faculty of „Carl Gustav Carus‟ TU-Dresden (EK27022006). Isolated T cells were cultured in RPMI 1640 complete medium supplemented with 200 U/ml IL-2 (Proleukin® S, Novartis Pharmaceuticals, Horsham, UK), 5 ng/ml IL-7 and 5 ng/ml IL-15 (ImmunoTools, Friesoythe, Germany) at densities of 1-2×10^6^ cells/ml. Production of lentiviral particles and transduction of primary human T cells was performed as described previously^8^. Briefly, T cells were activated with anti-CD3/CD28 coated polyclonal T cell activator beads (Thermo Fisher Scientific, Waltham, MA, USA) at beads to cell ratio of 1:4. Concentrated lentiviral vector supernatant was added 24h later and five additional times for the next 48h before beads removal. Throughout genetic modification and expansion, T cells were maintained in RPMI supplemented with cytokines. After 3 to 4 days in culture, cells were subsequently sorted using a FACSAria II (BD Biosciences). Isolated T cells were rested in RPMI supplemented with cytokines for additional 5 to 6 days. Media was replaced 24h before the assays by complete RPMI without recombinant cytokines^8^.

#### T cell activation and cytokine-release assay

For activation and cytokine release assays, modified T cells were co-cultured with target cells and TM. After 24h of incubation, supernatants were collected and T cells were stained to assess expression of PD-1 and CD69 markers using MACSQuant Analyzer^®^ (Miltenyi Biotec GmbH). Cytokine concentrations were determined by MACSPlex assay using the MACSPlex Cytokine 12 Kit (Miltenyi Biotec GmbH), MACSQuant Analyzer^®^ (Miltenyi Biotec GmbH) and MACSQuantify^®^ software (Miltenyi Biotec GmbH) according to the manufacturer’s instructions. In addition, enzyme-linked immunosorbent assay (ELISA) was also performed to confirm selected bead ELISA data, as previously described^5^.

#### Flow cytometry analysis

Isolated T cells were stained using fluorochrome labeled mAbs directed against human CD8 (BW135/80), CD279 (PD-1) (PD1313), CD4 (VIT4) and CD69 (FN50) acquired from Miltenyi Biotec. Staining of peripheral blood mononuclear cells (PBMCs) was performed using mAbs directed against human CD8 (BW135/80), CD19 (LT19), CD4 (VIT4) and CD56 (AF12-7H3) purchased from Miltenyi Biotec, and anti-STn mAbs L2A5 (ref. 27), B72.3 (ref. 36) and 3F1. Human Fc receptors expressed in PBMCs were blocked using FcR Blocking Reagent (Miltenyi Biotec) for 10 min at 4^o^C prior to staining, as described below. Binding of anti-STn TM to STn-expressing cancer cell lines and respective *K*_D_ was also assessed by flow cytometry analysis. Briefly, 3×10^5^ cells were incubated with 25 ng/μl or increasing concentrations of the α-STn TM for 1h followed by a washing step and subsequent incubation with mouse anti-La E5B9 mAb directed against the UniCAR-tag of the TM for 30 min. After washing, detection antibody PE-labeled goat anti-mouse IgG (Beckmann Coulter, Krefeld, Germany) was added for an incubation time of 30 min^11^. Alternatively, a similar procedure was performed for staining using anti-STn mAbs, in which 5 μg/μl of antibody was used followed by washing steps and further detection using PE-labeled goat anti-mouse IgG. Staining was analyzed using a MACSQuant^®^ Analyzer and MACSQuantify^®^ software (Miltenyi Biotec GmbH). Relative mean fluorescence intensities (MFI) of stained cells were analysed with GraphPad Prism 6 software (GraphPad Software Inc., La Jolla, CA, USA) and *K*_D_ value of one site specific binding was calculated from the resulting binding curve.

#### Cytotoxicity assay

T-cell mediated cell lysis was assessed by standard chromium release assay as previously described^12^. Briefly, modified T cells were co-cultured with [^51^Cr]-labeled STn-expressing cancer cells for 24h, in the presence or absence of TMs at the indicated concentrations and effector to target cells (E:T) ratios. For estimation of the maximum release samples containing the target cells were solubilized by 2.5% Triton X-100. Statistical analysis of mean specific lysis was performed by Student’s t-test.

#### Optical, small animal PET imaging and biodistribution of tumor xenograft models

All animal experiments were carried out at the Helmholtz-Zentrum Dresden-Rossendorf (HZDR) according to the guidelines of German Regulations for Animal Welfare and have been approved by the Landesdirektion Dresden (24-9165.40-4, 24.9168.21- 4/2004-1). Four weeks old female naïve athymic nude NMRI-Foxn1^nu/nu^ mice were purchased from JANVIER LABS (St. Berthevin, France). The mice were not randomized nor blinded. General anesthesia was induced with 10% (v/v) and maintained with inhalation of 8% (v/v) desflurane (Suprane, Baxter, Germany) in 30/10% (v/v) oxygen/air. For optical imaging analysis, 1.5×10^6^ MDA-MB-231 STn-Luc cells were mixed with 1×10^6^ UniCAR 28/ζ T cells in the presence or absence of 10 µg of anti-STn TM. Respective mixtures were injected subcutaneously in the right thigh of experimental mice. Luminescence imaging of anesthetized mice was performed 10min after i.p. injection of 200 µL of D-luciferin potassium salt (15 mg/mL) (Thermofisher, Dreieich, Germany) starting at day 0 and followed at day 1, 3 and 6 using a dedicated small animal multimodal imaging system (Xtreme, Bruker, Germany).

For immuno-PET imaging and biodistribution analysis, mice were inoculated subcutaneously in the right hind flank with 1.5×10^6^ MDA STn-Luc cells in PBS. Three to four weeks after cell inoculation animals bearing tumors between 100 and 500 mm^3^ as measured by a caliper and visual inspection were selected for PET or biodistribution studies. To evaluate tumor targeting, the TMs were conjugated with NODAGA (NODAGA-anti-STn TM) and further labeled with the PET isotope ^64^Cu^2+^ ([^64^Cu]Cu-NODAGA-anti-STn TM) according to standard procedures^13,14^ Immuno-PET imaging was performed in three MDA-MB-231 STn-Luc tumor bearing mice (30.9 ± 5 g). [^64^Cu]Cu-NODAGA-anti-STn TM (12.0 ± 3.1 MBq containing 0.6 nmol anti-STn TM) was intravenously injected into a lateral tail vein of the mice. Dynamic scans were acquired over 2h and a static scan with a duration of one hour was carried out 13h after injection using a small animal PET/CT scanner (NanoPET/CT, Mediso). Activity concentrations were expressed as standard uptake values (SUV), which is the activity concentration in the tissue normalized to the body weight, that is defined as tissue concentration (MBq/mL) /injected dose (MBq) /body weight (g) in (g/mL). To characterize the target to background ratio (TBR) the tumor to muscle and tumor to blood ratios were calculated. The distribution of the activity amounts measured in complete organs was expressed as percentage of injected activity (%ID). Images were visualized and quantified using ROVER software (ABX GmbH, Germany).

To evaluate the biodistribution of [^64^Cu]Cu-anti-STn TM, four MDA-MB-231 STn-Luc tumor bearing mice (body weight 23.0 ± 0.8 g) obtained a single intravenous injection of the radiotracer (0.54 ± 0.2 MBq) with a specific activity of 21.3 GBq/µmol corresponding to 13 pmol anti-STn TM per animal. Two hours after injection the mice were sacrificed, blood, selected organs and tissues were taken, dried, weighted and measured in cross-calibrated calibrator γ-well-counter. Quantitative data were calculated in comparison to standard samples and expressed as percentage injected dose (%ID) and SUV.

#### Statistical analysis

Statistical analysis was performed with GrapPad Prism software version 6.0 (GraphPad Software Inc., La Jolla, CA, USA).

### REFERENCES

1 Ferreira JA, Videira PA, Lima L, Pereira S, Silva M, Carrascal M *et al.* Overexpression of tumour-associated carbohydrate antigen sialyl-Tn in advanced bladder tumours. *Mol Oncol* 2013; **7**: 719–731.

2 Julien S, Krzewinski-Recchi MA, Harduin-Lepers A, Gouyer V, Huet G, Le Bourhis X *et al.* Expression of Sialyl-Tn antigen in breast cancer cells transfected with the human CMP-Neu5Ac: GalNAc α2,6-sialyltransferase (ST6GalNAc I) cDNA. *Glycoconj J* 2001; **18**: 883–893.

3 Arndt C, Feldmann A, Bonin M Von, Cartellieri M, Ewen E, Koristka S *et al.* Costimulation improves the killing capability of T cells redirected to tumor cells expressing low levels of CD33 : description of a novel modular targeting system. 2014; : 59–69.

4 Arndt C, Feldmann A, Koristka S, Cartellieri M, Dimmel M, Ehninger A *et al.* Simultaneous targeting of prostate stem cell antigen and prostate-specific membrane antigen improves the killing of prostate cancer cells using a novel modular T cell-retargeting system. *Prostate* 2014; **74**: 1335–1346.

5 Feldmann A, Arndt C, Topfer K, Stamova S, Krone F, Cartellieri M *et al.* Novel Humanized and Highly Efficient Bispecific Antibodies Mediate Killing of Prostate Stem Cell Antigen-Expressing Tumor Cells by CD8+ and CD4+ T Cells. *J Immunol* 2012; **189**: 3249–3259.

6 Arndt C, Feldmann A, Töpfer K, Koristka S, Cartellieri M, Temme A *et al.* Redirection of CD4 ^+^ and CD8 ^+^ T lymphocytes via a novel antibody-based modular targeting system triggers efficient killing of PSCA ^+^ prostate tumor cells. *Prostate* 2014; **74**: 1347–1358.

7 Cartellieri M, Feldmann A, Koristka S, Arndt C, Loff S, Ehninger A *et al.* Switching CAR T cells on and off: a novel modular platform for retargeting of T cells to AML blasts. *Blood Cancer J* 2016; **6**: e458.

8 Cartellieri M, Koristka S, Arndt C, Feldmann A, Stamova S, Von Bonin M *et al.* A novel Ex Vivo isolation and expansion procedure for chimeric antigen receptor engrafted human T cells. *PLoS One* 2014; **9**. doi:10.1371/journal.pone.0093745.

9 Bippes CC, Feldmann A, Stamova S, Cartellieri M, Schwarzer A, Wehner R *et al.* A novel modular antigen delivery system for immuno targeting of human 6-sulfo LacNAc-positive blood dendritic cells (slanDCs). *PLoS One* 2011; **6**. doi:10.1371/journal.pone.0016315.

10 Szymczak AL, Workman CJ, Wang Y, Vignali KM, Dilioglou S, Vanin EF *et al.* Correction of multi-gene deficiency in vivo using a single ‘self-cleaving’ 2A peptide-based retroviral vector. *Nat Biotechnol* 2004; **22**: 589–594.

11 Koristka S, Cartellieri M, Arndt C, Bippes CC, Feldmann A, Michalk I *et al.* Retargeting of regulatory T cells to surface-inducible autoantigen La/SS-B. *J Autoimmun* 2013; **42**: 105–116.

12 Feldmann A, Stamova S, Bippes CC, Bartsch H, Wehner R, Schmitz M *et al.* Retargeting of TCells to Prostate Stem Cell Antigen ExpressingT umor Cells : Comparison of Different Antibody Formats. 2011; **1011**: 998–1011.

13 Albert S, Arndt C, Feldmann A, Bergmann R, Bachmann D, Koristka S *et al.* A novel nanobody-based target module for retargeting of T lymphocytes to EGFR-expressing cancer cells via the modular UniCAR platform. *Oncoimmunology* 2017; **6**. doi:10.1080/2162402X.2017.1287246.

14 Bergmann R, Albert S, Feldmann A, Berdnt N, Bachmann M. The UniCAR system: Biodistribution and kinetics of the 64Cu-labeled anti EGFR target module in mice. *J Nucl Med* 2017; **58**.
