# Supplementary figures and images for: Development of a novel target module redirecting UniCAR T cells to Sialyl Tn-expressing tumor cells
Source: Blood Cancer J. 2018 Aug 22;8(9):81. doi: 10.1038/s41408-018-0113-4 (PMC6127150; doi:10.1038/s41408-018-0113-4)

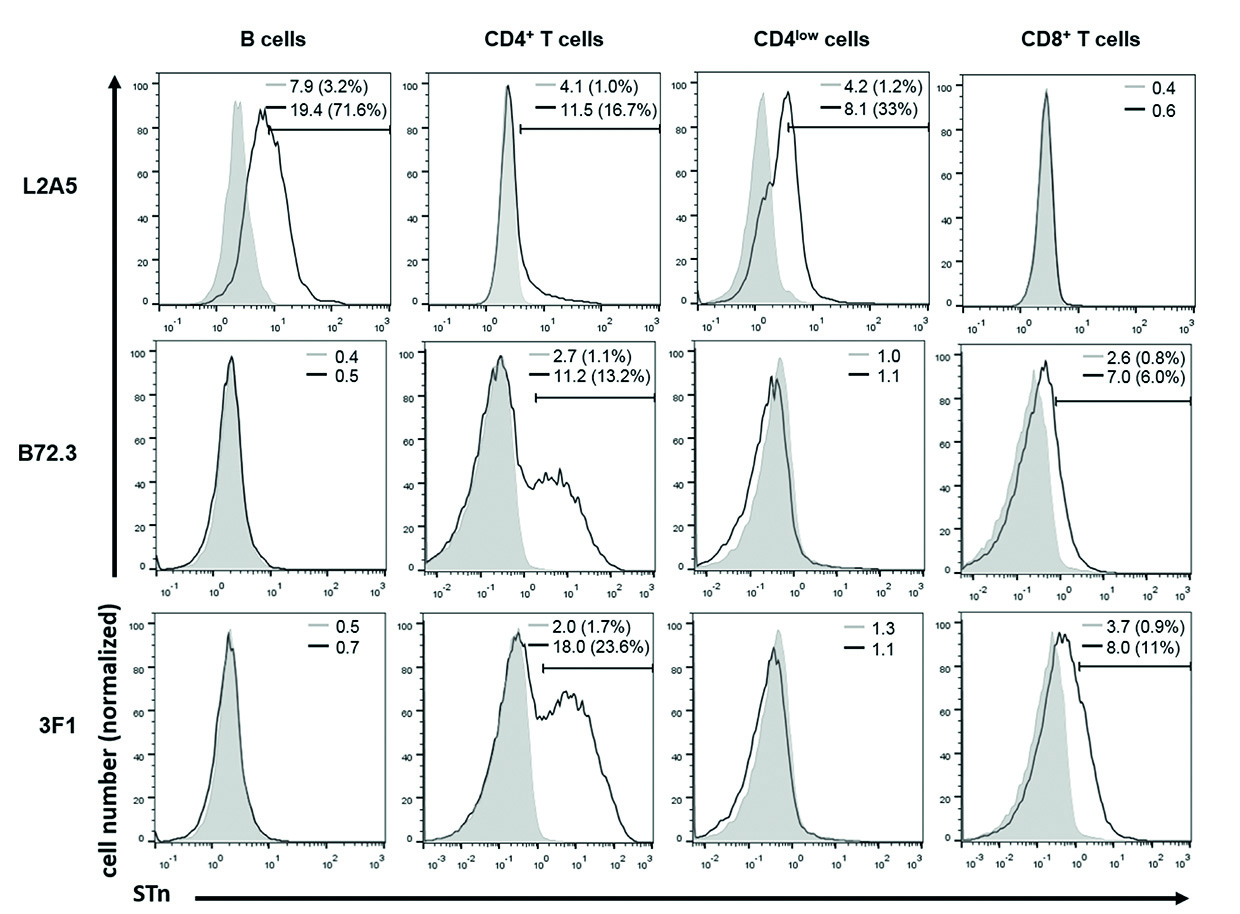

Supplement: Supplementary file 2 — Supplementary Figure 1 [file 41408_2018_113_MOESM2_ESM.jpg]

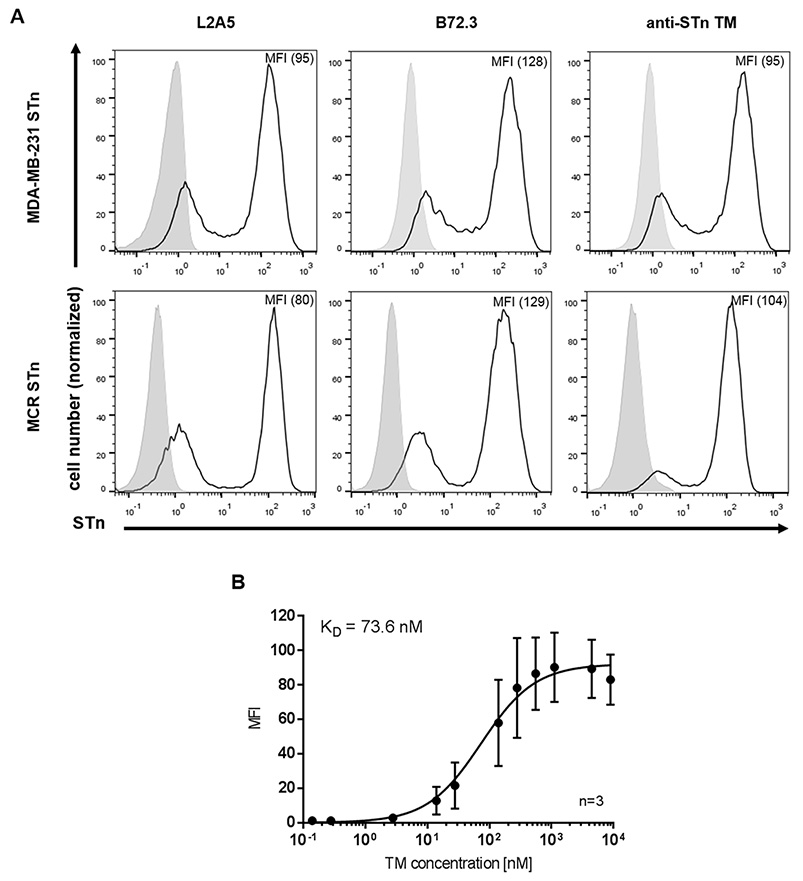

Supplement: Supplementary file 5 — Supplementary Figure 2 [file 41408_2018_113_MOESM5_ESM.jpg]

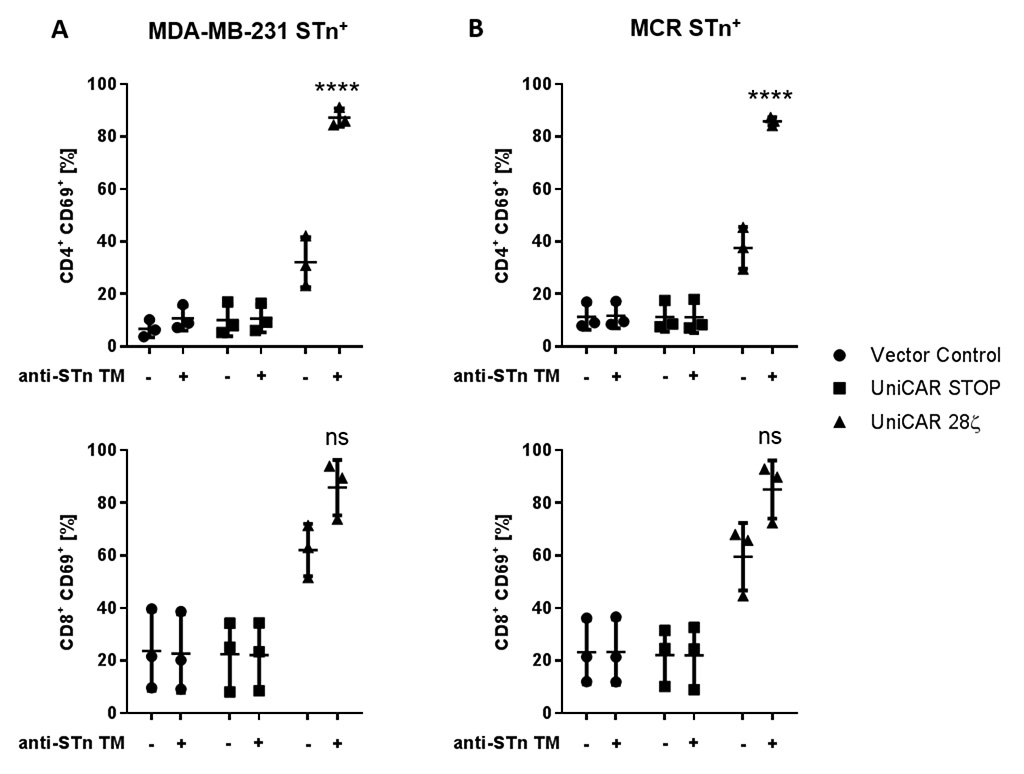

Supplement: Supplementary file 8 — Supplementary Figure 3 [file 41408_2018_113_MOESM8_ESM.jpg]
