# Supplementary material for: Development of a novel target module redirecting UniCAR T cells to Sialyl Tn-expressing tumor cells
Source: Blood Cancer J. 2018 Aug 22;8(9):81. doi: 10.1038/s41408-018-0113-4 (PMC6127150; doi:10.1038/s41408-018-0113-4)
Supplement: Supplementary file 3 — Figure Legend Suppl. Fig. 1 [file 41408_2018_113_MOESM3_ESM.docx]

**Supplementary Fig 1. Binding assessment of anti-STn mAbs to PBMCs.** Freshly isolated PBMCs from healthy donors were obtained as described in Supplementary Materials and Methods and further stained by incubating with the anti-STn mAbs L2A5, B72.3 and 3F1 for 1h at 4°C. Binding of the respective mAb was detected using PE-labeled goat anti-mouse IgG. To distinguish different leukocyte subsets, cells were stained using the cell surface markers CD4 and CD8 (T cells), and CD19 (B cells). Numbers represent percentage of positive cells or MFI of total cells under the marker for each condition. Isotype control represented as filled gray area. Results of one representative donor are shown.
