# Supplementary material for: Development of a novel target module redirecting UniCAR T cells to Sialyl Tn-expressing tumor cells
Source: Blood Cancer J. 2018 Aug 22;8(9):81. doi: 10.1038/s41408-018-0113-4 (PMC6127150; doi:10.1038/s41408-018-0113-4)
Supplement: Supplementary file 4 — Supplementary Figure 1 Text summary [file 41408_2018_113_MOESM4_ESM.docx]

The supplementary information herein given represents data as a set of histograms in a figure.

**Supplementary Fig 1.** shows the binding analysis of the anti-STn mAbs L2A5, B72.3 and 3F1 to PBMCs. This provides evidence and detailed information regarding the binding of such antibodies to subpopulations of immune cells.
