# Supplementary material for: Development of a novel target module redirecting UniCAR T cells to Sialyl Tn-expressing tumor cells
Source: Blood Cancer J. 2018 Aug 22;8(9):81. doi: 10.1038/s41408-018-0113-4 (PMC6127150; doi:10.1038/s41408-018-0113-4)
Supplement: Supplementary file 6 — Figure Legend Suppl. Fig. 2 [file 41408_2018_113_MOESM6_ESM.docx]

**Supplementary Fig 2. Analysis of anti-STn TM binding to STn-expressing cancer cells. (A)** STn-expressing MDA-MB-231 or MCR cancer cells (3×10^5^ cells) were incubated with 25 ng/μl or increasing concentrations of the α-STn TM for 1h, followed by detection using the anti-La mAb E5B9 directed against the UniCAR epitope and PE-conjugated goat anti-mouse IgG for 30 min. As positive controls, cells were stained with anti-STn mAbs L2A5 or B72.3 followed by detection with PE-conjugated anti-mouse-IgG mAb. Histograms display cells stained with each control mAb or with anti-STn TM (black line curves). **(B)** For estimation of the K_D_ value, increasing amounts of anti-STn TM were used to stain MDA-MB-231 STn-expressing cells followed by detection using the anti-La mAb E5B9 directed against the UniCAR epitope and PE-conjugated anti-mouse-IgG mAb. The K_D_ value was calculated from the resulting binding curve using GraphPad Prism 6 software. Results from three individual donors are presented.
