# Supplementary material for: Development of a novel target module redirecting UniCAR T cells to Sialyl Tn-expressing tumor cells
Source: Blood Cancer J. 2018 Aug 22;8(9):81. doi: 10.1038/s41408-018-0113-4 (PMC6127150; doi:10.1038/s41408-018-0113-4)
Supplement: Supplementary file 7 — Supplementary Figure 2 Text summary [file 41408_2018_113_MOESM7_ESM.docx]

The supplementary information herein given represents data as a set of histograms and plot in a figure.

**Supplementary Fig 2.** shows the binding profile of the anti-STn mAbs L2A5 and B72.3, and the anti-STn TM to STn-expressing MDA-MB-231 and MCR cancer cells. Estimation of the K_D_ value was further performed using a titration curve. This provides evidence and detailed information regarding the binding of the anti-STn TM to different types of cancer cells, and the respective binding affinity.
