# Supplementary material for: Development of a novel target module redirecting UniCAR T cells to Sialyl Tn-expressing tumor cells
Source: Blood Cancer J. 2018 Aug 22;8(9):81. doi: 10.1038/s41408-018-0113-4 (PMC6127150; doi:10.1038/s41408-018-0113-4)
Supplement: Supplementary file 9 — Figure Legend Suppl. Fig. 3 [file 41408_2018_113_MOESM9_ESM.docx]

**Supplementary Fig 3. Antigen-dependent activation of UniCAR T cells using STn-specific TM.** Human T cells from healthy donors were transduced with lentiviral vectors encoding only the EGFP marker protein (circles), UniCAR constructs lacking intracellular signaling domains (squares) or containing a dual CD28/CD3ζ signaling domain (triangles), as described in Supplementary Materials and Methods. The respective genetically engineered T cells were incubated for 24h with STn-expressing tumor cell lines MDA-MB-231 **(A)** or MCR **(B)** in the presence or absence of 80 nM anti-STn TM. After incubation, cells were harvested and stained for CD4, CD8 and CD69 surface expression. Upper and lower panel plots show the analysis of the activation markers CD69 for CD4^+^ T cells and CD8^+^ T cells, respectively. Results from three individual donors are presented as mean ± SD. Statistical analysis was performed using two-way ANOVA with Bonferroni multiple-comparison test (ns = not significant; ****p<0.0001).
