# Supplementary material for: Development of a novel target module redirecting UniCAR T cells to Sialyl Tn-expressing tumor cells
Source: Blood Cancer J. 2018 Aug 22;8(9):81. doi: 10.1038/s41408-018-0113-4 (PMC6127150; doi:10.1038/s41408-018-0113-4)
Supplement: Supplementary file 10 — Supplementary Figure 3 Text summary [file 41408_2018_113_MOESM10_ESM.docx]

The supplementary information herein given represents data as a set of plots in a figure.

**Supplementary Fig 3.** shows the activation profile of CD4 and CD8 T cells for the anti-STn TM using the STn-expressing MDA-MB-231 and MCR cancer cell lines, based on the activation marker CD69. This provides evidence and detailed information regarding the specific activation of UniCAR T cells using the anti-STn TM in different types of cancer cell lines.
